# Chinese Herbal Medicine Combined with Conventional Therapy for Blood Pressure Variability in Hypertension Patients: A Systematic Review of Randomized Controlled Trials

**DOI:** 10.1155/2015/582751

**Published:** 2015-05-18

**Authors:** Zhuo Chen, Liqiong Wang, Guoyan Yang, Hao Xu, Jianping Liu

**Affiliations:** ^1^Graduate School, Beijing University of Chinese Medicine, Beijing 100029, China; ^2^Centre for Evidence-Based Chinese Medicine, Beijing University of Chinese Medicine, Beijing 100029, China; ^3^Cardiovascular Diseases Center, Xiyuan Hospital, China Academy of Chinese Medical Sciences, Beijing 100091, China

## Abstract

*Objective*. The aim of this systematic review is to evaluate effect of Chinese medicine combined with conventional therapy on blood pressure variability (BPV) in hypertension patients. *Methods*. All randomized clinical trials (RCTs) comparing Chinese medicine with no intervention or placebo on the basis of conventional therapy were included. Data extraction, analyses, and quality assessment were performed according to the Cochrane standards. *Results*. We included 13 RCTs and assessed risk of bias for all the trials. Chinese medicine has a significant effect in lowering blood pressure (BP), reducing BPV in the form of standard deviation (SD) or coefficient of variability (CV), improving nighttime BP decreased rate, and reversing abnormal rhythm of BP. *Conclusions*. Chinese medicine was safe and showed beneficial effects on BPV in hypertension patients. However, more rigorous trials with high quality are warranted to give high level of evidence before recommending Chinese medicine as an alternative or complementary medicine to improve BPV in hypertension patients.

## 1. Background

It is estimated that there are nearly one billion people suffering from hypertension worldwide, and the number of patients will increase to 1.5 billion by 2050 [1]. Blood pressure variability (BPV) means the degree of blood pressure (BP) fluctuations in a certain period of time. BPV is regarded as a separate index which is different from the BP reflecting cardiovascular activities. Many studies have confirmed that BPV could impact on hypertensive target organ damage and overall prognosis of patients [1–5]. An important factor to improve the prognosis of hypertensive patients is to reduce the BPV effectively. Clinicians pay close attention to lowering pressure steadily nowadays. There have been many studies that elucidated the relationship between western medicine and BPV. Although the results are inconclusive, most studies have shown that calcium channel blockers (CCB) are the most effective to reduce the BPV, especially amlodipine. Amlodipine in combination with other drugs may be more effective [6–8].

Short-term BPV within the 24-hour period is easier to obtain measured results. Ambulatory blood pressure monitoring (ABPM) is a way to assess the short-term BPV at present. By ABPM, a large amount of cross-sectional studies confirmed the increased BPV indicating the aggravated target organ damage [9]. A quantitative analysis of 155 Chinese people's ABPM results showed that BPV of hypertensive patients was higher than that of healthy people [10].

Western medicine puts emphasis on quickly and effectively lowering BP and helps patients reach target BP as soon as possible. But even effective long-term control of BP by western medicine may not fully achieve goals of protecting target organs against damage, because reversing the target organ damage is a long process. Chinese medicine highlights the overall concept, self-regulation mechanism, and multitargets action and often plays an important role in the protection of target organ of hypertensive patients [11]. However, whether Chinese medicine is effective on BPV remains unclear. So, we design this systematic review to evaluate effect of Chinese medicine on BPV by ABPM in hypertension patients.

## 2. Methods

### 2.1. Search Strategy

Two reviewers (Z. Chen and L. Q. Wang) searched the following databases from their inception to April 15, 2014, for the identification of randomized clinical trials (RCTs) assessing Chinese herbal medicine for BPV: The Cochrane Library, Pubmed, China National Knowledge Infrastructure (CNKI), Chinese Scientific Journal Database (VIP), Wanfang Databases, and Sino-Med Database. There was no limitation in languages. We also searched references of included reviews, just in case eligible trials were missed. We searched using the following searching terms: “blood pressure variability,” “BPV,” “essential hypertension,” “standard deviation,” “coefficient of variability,” “traditional Chinese medicine,” “Chinese herbal medicine,” and “randomized controlled trial.” Chinese pinyin of these terms were “xue ya bian yi,” “xue ya bo dong,” “xue ya bian yi xing,” “xue ya bo dong xing,” “zhong yao,” “zhong chengyao,” “zhong cao yao,” “zhong xi yi,” “ke li,” “zhong yi yao,” “zhong yi,” and “sui ji.” Based on different characteristics of literature databases, we adapted the search strategies appropriately which are shown in Table 1.

### 2.2. Inclusion Criteria

#### 2.2.1. Study Design

RCTs were included regardless of blind method and language.

#### 2.2.2. Participants

Patients with BPV regardless of age and race were included, who were diagnosed by one of the following diagnostic criteria: WHO/ISH Hypertension Prevention Guide (1999 [12]/2003 [13]) or Chinese Hypertension Prevention Guide (2005 [14]/2010 [15]). Patients with severe liver and kidney dysfunction and serious complications were excluded.

#### 2.2.3. Interventions

Chinese herbal medicine was used as intervention in at least one group of the study, form of which can be decoction, granule, and Chinese patent medicine (capsule/tablet). The method of application was restricted to orally taken; therefore, injections were excluded. Control groups were no treatment, placebo, or conventional medicines. Trials of Chinese herbal medicine in combination of conventional medicine compared to conventional medicine alone were also included, if the conventional medicine applied in both groups was the same. Trials with a treatment course of less than four weeks were excluded. Trials with more than one kind of Chinese herbal medicine as intervention were also excluded.

#### 2.2.4. Outcome Measures

Primary outcome measures were BPV (measured by standard deviation (SD) and coefficient of variability (CV), at three different time points: 24 hours, day, and night), BP (24 hours, day, and night), and symptom improvement rate which had uniform criteria, that is, clinical guideline of new drugs for traditional Chinese medicine. Among all these indicators, BPV and ambulatory BP are both indispensable. Secondary outcome measures were adverse events, quality of life (QOL), the abnormal rhythm of BP reverse rate, and nighttime BP decrease rate.

### 2.3. Data Extraction and Quality Assessment

**Figure 1:**
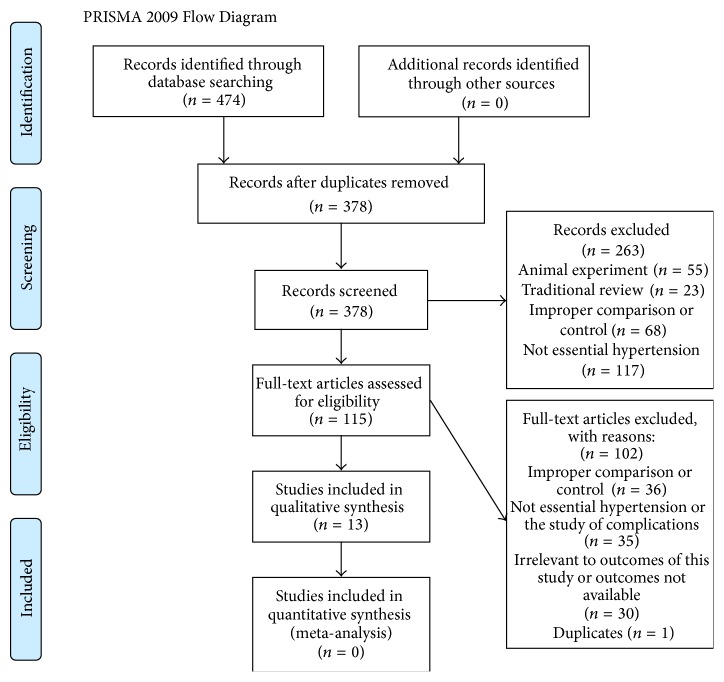
Flow chart of study selection.

Two authors (Z. Chen and L. Q. Wang) performed data extraction (Figure 1) independently according to a predesigned form. Disagreements during cross-checking of the data extraction form were resolved by consensus or consultation from a third author (J. P. Liu). We assessed the methodological quality of these trials by risk of bias tool which was recommended by Cochrane Handbook [16]. Specific items of the risk of bias tool were as follows: selection bias (random sequence generation, allocation concealment), performance bias (blinding of participants and personnel), detection bias (blinding of outcome assessment), attrition bias (incomplete outcome data), reporting bias (selective outcome reporting), and other biases. Each item for all included trials was judged into “high risk,” “unclear,” and “low risk.”

### 2.4. Data Analysis and Synthesis

Revman 5.2 software provided by the Cochrane Collaboration was used for data analyses. We expressed dichotomous data as risk ratio (RR) and its 95% confidence intervals (CI) and continuous outcome as mean difference (MD) and its 95% CI. Since all included trials applied different interventions, we did not pool the data due to the clinical heterogeneity. We performed qualitative description of the data synthesis.

We also failed to conduct a funnel plot to explore publication bias, because the number of included studies was less than nine for each outcome.

## 3. Results

### 3.1. Description of Included Trials

474 trials were identified from six databases. Among them, 96 records were removed because of duplicates. By screening titles and abstracts, we excluded 263 records for reasons of animal experiment, traditional review, improper comparison, or nonprimary hypertension. By browsing full-text article, we excluded 102 records for reasons of improper comparison, nonprimary hypertension, complications, uncorrelated outcomes, or duplicate publication. At last, a total of 13 articles [17–29] that met inclusion criteria were included into this systematic review. (Basic characteristics of included studies are presented in Table 2.)

#### 3.1.1. Study Characteristics

The 13 trials were published in Chinese from 2007 to 2013, of which seven [17–20, 27–29] were academic dissertations, five [21–25] were journal articles, and one [26] was conference paper. All trials were carried out in mainland China. One article [21] was funded by Shandong province science and technology development project.

#### 3.1.2. Population Characteristics

1103 patients were included, with an average of 85 cases per trial (range from 40 cases to 160 cases). Eight trials [17–21, 25–27] included both inpatients and outpatients, three trials [21, 23, 29] only contained outpatients, and two trials [24, 28] did not report whether outpatients or inpatients. All trials claimed baseline characteristics were comparable between the groups. Five trials [17, 23, 24, 26, 29] did not report male/female ratio in different groups, three trials [24, 26, 28] did not report distribution of age, and five [21, 23, 24, 26, 29] papers did not report disease duration between the groups. The remaining trials clearly reported these items. High BP classification was limited to first and second degree, except two trials (one [24] did not report high BP classification and one [25] was not limited classification). One trial [17] explicitly mentioned complications, but there was no significant difference in the distribution and two groups can be comparable. Remaining trials did not report complications.

#### 3.1.3. Comparisons

There were two types of comparisons: (1) Chinese medicine combined with conventional therapy versus conventional therapy alone (twelve trials) and (2) Chinese medicine combined with conventional therapy versus placebo and conventional therapy (one trial). Conventional therapy of six trials [17, 20–22, 26, 29] was amlodipine besylate tablet 5 mg per day. One trial was levamlodipine [28]. One trial was nifedipine controlled released tablet [27]. Two trials [18, 24] were CCB combined with ACEI. Two trials were ACEI [19, 23]. One trial did not report the ingredients of Chinese medicine [25]. There were four dosage forms: decoction, capsule, granule, and tablet. (Herbal medicines and adverse effects in the included trials are presented in Table 4.)

#### 3.1.4. Outcome Measures

Nine trials [17, 19, 22, 24–29] reported adverse events. Two trials [20, 23] clearly reported there was no adverse drug reaction. Two trials [23, 29] reported QOL. No trial reported health-economic indicators or follow-up visit. All trials reported BPV in the form of SD or CV, symptom improvement rate (antihypertensive effect) of which diagnostic criterion was Clinical Research Guideline of New Drugs for Traditional Chinese Medicine. Antihypertensive effect is divided into three levels according to the BP value: markedly effective, effective, and invalid.

### 3.2. Methodological Quality

**Figure 2:**
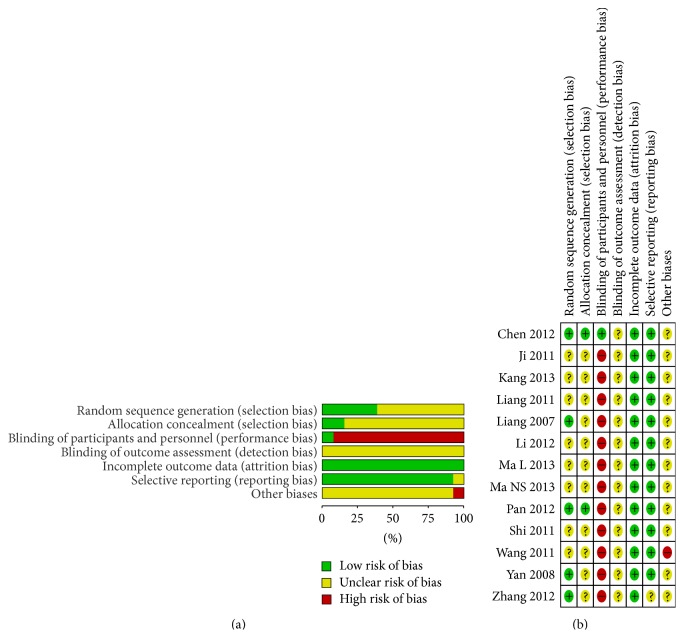
(a) Risk of bias graph: review authors' judgments about each risk of bias item presented as percentages across all included studies. (b) Risk of bias summary: review authors' judgments about each risk of bias item for each included study. “+”: low risk of bias; “?”: unclear risk of bias; or “−” high risk of bias.

Five trials [17, 19, 26, 27, 29] used random number table to generate the random sequence. Two trials [17, 29] referred to opaque sealed envelopes. Blinding of participants and personnel was mentioned in only one trial [29] which was double-blinding. No trial blinded the outcome assessors. There was not sufficient information to judge whether outcome assessors were blinded or not. Eleven trials did not miss outcome data, among which two trials [17, 26] clearly claimed no drop-out patient. All trials reported their prespecified primary outcomes except one trial. All trials declared baseline characteristics were comparable. One trial [21] was lack of the inclusion and exclusion criteria. Unfortunately, no trial reported sample size calculation. (Risk of bias summaries are presented in Figure 2.)

### 3.3. Effects of Interventions

Since every trial had different Chinese herbal medicines as treatment, none of trials could be analyzed by meta-analysis, because of clinical heterogeneity. We presented the effects of interventions by qualitative description, according to the two types of comparisons: Chinese medicine combined with conventional therapy versus the same conventional therapy and Chinese medicine combined with conventional therapy versus placebo combined with the same conventional therapy. All BPV were measured by ambulatory BP meter. BPV mainly was expressed in two forms: SD and CV. (Effect estimate of outcomes is presented in Table 3.)

#### 3.3.1. Chinese Medicine Combined with Conventional Therapy versus the Same Conventional Therapy

*(1) SD after Treatment*. There were nine trials [17, 18, 20, 22–24, 26–29] reporting 24 h systolic SD. Five trials [20, 22–24, 26] found statistical difference between groups. The results have shown that combination therapy is superior to conventional treatment. For traditional Chinese medicine (TCM) combined with amlodipine besylate tablet, three trials showed a reduction of 24 h systolic SD: Sangji Wendan decoction (MD −2.44; 95% CI −4.38 to −0.50; *n* = 40), Xiandan Tongmai decoction (MD −2.40; 95% CI −3.50 to −1.30; *n* = 118), and Yin gan jing decoction (MD −0.90; 95% CI −1.45 to −0.35; *n* = 80). Xiang tian ma decoction combined with benazepril also reduced 24 h systolic SD (MD −5.20; 95% CI −6.37 to −4.03; *n* = 78). Xuezhikang capsule was integrated with angiotensin converting enzyme inhibitors (ACEI) and calcium channel blockers (CCB) (MD −1.79; 95% CI −2.79 to −0.79; *n* = 110). There were no significant differences in the other four trials; they were Tianma Shuxin granule combined with amlodipine besylate tablet, Qianyang Yuyin granule combined with felodipine and enalapril, Tiao ping kang tablet combined with nifedipine controlled released tablets, and Gouteng Siwu decoction combined with levamlodipine besylate tablet.

Nine trials [17, 18, 20, 22–24, 26–29] reported 24 h diastolic SD. Two of them which used amlodipine besylate tablet as control group found a significant effect in lowering 24 h diastolic SD in the experimental group superior to that in the control group, that is Sangji Wendan decoction (MD −2.13; 95% CI −4.34 to 0.08; *n* = 40) and Yin gan jing decoction (MD −0.24; 95% CI −0.46 to −0.02; *n* = 80). The other seven trials did not find significant difference between groups: Xiandan Tongmai decoction combined with amlodipine besylate tablet, Tianma Shuxin granule combined with amlodipine besylate tablet, Xiang tian ma decoction combined with benazepril, Xuezhikang capsule integrated with ACEI and CCB, Qianyang Yuyin granule combined with felodipine and enalapril, Tiao ping kang tablet combined with nifedipine controlled released tablets, and Gouteng Siwu decoction combined with levamlodipine besylate tablet.

Day systolic SD was reported by nine trials. Five trials [17–23, 25, 27] significantly lowered this outcome in experimental group superior to that in control group. They were Qin dan capsule plus enalapril maleate tablet (MD −2.15; 95% CI −3.39 to −0.91; *n* = 61), Xiandan Tongmai decoction plus amlodipine besylate tablet (MD −1.40; 95% CI −2.63 to −0.17; *n* = 118), Xiang tian ma decoction plus benazepril (MD −4.56; 95% CI −5.85, −3.27; *n* = 78), Yangxue Qingnao granule plus conventional antihypertensive drugs (MD −0.50; 95% CI −0.81 to −0.19; *n* = 160), and Tiao ping kang tablet plus nifedipine controlled released tablets (MD −3.87; 95% CI −6.16 to −1.58; *n* = 58). The other four trials did not find significant difference between groups, one trial about Qianyang Yuyin granule plus felodipine with enalapril and three trials compared amlodipine besylate tablets, that were Tianma Shuxin granule, Sangji Wendan decoction, and Songling Xuemaikang capsule.

Day diastolic SD was reported by nine trials [17–23, 25–27]. Five trials found the effect of lowering this outcome in experimental group superior to that in control group. They were Qin dan capsule combined with enalapril maleate tablet (MD −2.20; 95% CI −3.26 to −1.14; *n* = 61), Songling Xuemaikang capsule combined with amlodipine besylate tablet (MD −0.60; 95% CI −1.08 to −0.12; *n* = 138), Xiandan Tongmai decoction combined with amlodipine besylate tablet (MD −1.90; 95% CI −3.28 to −0.52; *n* = 118), Xiang tian ma decoction combined with benazepril (MD −1.14; 95% CI −2.09 to −0.19; *n* = 78), and Tiao ping kang tablet combined with nifedipine controlled released Tablets (MD −4.95; 95% CI −7.10 to −2.80; *n* = 58). Although the other four trials had reported this outcome too, there was no statistically significant difference. The four trials were Tianma Shuxin granule combined with amlodipine besylate tablet, Qianyang Yuyin granule combined with felodipine and enalapril, Yangxue Qingnao granule combined with conventional antihypertensive drugs, and Sangji Wendan decoction combined with amlodipine besylate tablet.

Nine trials [17–23, 25, 27] reported night systolic SD. Six trials showed a reduction of night systolic SD. Two of them are compared with amlodipine besylate tablet; they were Songling Xuemaikang capsule (MD −0.60; 95% CI −1.08 to −0.12; *n* = 138) and Xiandan Tongmai decoction (MD −1.90; 95% CI −3.28 to −0.52; *n* = 118), besides, Xiang tian ma decoction combined with benazepril (MD −1.14; 95% CI −2.09 to −0.19; *n* = 78), Qin dan capsule combined with enalapril maleate tablet (MD −2.20; 95% CI −3.26 to −1.14; *n* = 61), Tiao ping kang tablet combined with nifedipine controlled released tablets (MD −4.95; 95% CI −7.10 to −2.80; *n* = 58), and Yangxue Qingnao granule combined with conventional antihypertensive drugs (MD −0.39; 95% CI −0.60 to −0.18; *n* = 160). Three trials showed no significant difference, that is, Tianma Shuxin granule combined with amlodipine besylate tablet, Qianyang Yuyin granule combined with felodipine and enalapril, and Sangji Wendan decoction combined with amlodipine besylate tablet.

There were nine trials [17–23, 25, 27] reporting night diastolic SD. Three trials showed significant reduction of this outcome: Xiang tian ma decoction combined with benazepril (MD −1.19; 95% CI −2.14 to −0.24; *n* = 78), Yangxue Qingnao granule combined with conventional antihypertensive drugs (MD −0.21; 95% CI −0.36 to −0.06; *n* = 160), and Qin dan capsule combined with enalapril maleate tablet (MD −1.40; 95% CI −2.51 to −0.29; *n* = 61). Results of the remaining six trials had no statistical differences. 

*(2) CV after Treatment*. Only one trial (Tianma Shuxin granule combined with amlodipine besylate tablet) reported CV. There were statistically significant differences in two outcomes. There was a reduction in day systolic CV in experimental group superior to that in control group (MD −0.01; 95% CI −0.02 to −0.00; *n* = 60). There was a reduction in 24 h diastolic CV in experimental group inferior to that in control group (MD 0.03; 95% CI 0.02 to 0.04; *n* = 60). Nevertheless, there was a reduction in day systolic CV in control group inferior to that in experimental group (MD −0.01; 95% CI −0.02 to −0.00; *n* = 60). There was no statistically significant advantage in reducing CV in experimental group in terms of 24 h systolic CV, day diastolic CV, night systolic CV and night diastolic CV. 

*(3) BP after Treatment*. Seven trials [18, 19, 21, 24–26, 28] presented that integrative medicine had the advantage in reducing 24 h systolic BP. Six trials [18, 21, 23, 25, 26, 28] presented that integrative medicine had the advantage in reducing 24 h diastolic BP. There were three trials [18, 25, 26] showing that integrative medicine had the advantage in reducing day systolic BP; however, one trial [23] had the opposite result. Four trials [23, 25–27] presented that integrative medicine had the advantage in reducing day diastolic BP. There were five trials [18, 23, 25–27] showing that integrative medicine had the advantage in reducing night systolic BP. Four trials [18, 25–27] presented that integrative medicine had the advantage in reducing night diastolic BP. 

*(4) Antihypertensive Effect*. Antihypertensive effect was measured taking clinical guideline of new drugs for TCM as standard. Only one trial [26] showed treatment group superior to control group in improving antihypertensive effect. 

*(5) QOL*. One trial [23] referred to QOL but did not report which specific test scale it used. 

*(6) Nighttime BP Decreased Rate*. Three trials [19, 26, 28] reported this outcome. Only one trial [26] presented that combination therapy is superior to conventional treatment in nighttime BP decreased rate of both systolic BP and diastolic BP. 

*(7) Frequency of Reversed Abnormal Rhythm of BP*. Two trials [25, 27] showed treatment group superior to control group in increasing frequency of reversed abnormal rhythm of BP.

#### 3.3.2. Chinese Medicine Combined with Conventional Therapy versus Placebo Combined with the Same Conventional Therapy

There was only one trial [29] under this category, that is, Qing xuan granule combined with amlodipine besylate tablet versus placebo combined with amlodipine besylate tablet. 

*(1) SD after Treatment*. All the outcomes about SD of this trial were of significant statistical heterogeneity. Qing xuan granule combined with amlodipine besylate tablet had a better efficacy than the control in terms of lowering 24 h systolic SD (MD −1.47; 95% CI −2.52 to −0.42; *n* = 90), 24 h diastolic SD (MD −1.26; 95% CI −1.93 to −0.59; *n* = 90), day systolic SD (MD −1.60; 95% CI −2.65 to −0.55; *n* = 90), day diastolic SD (MD −1.02; 95% CI −1.70 to −0.34; *n* = 90), night systolic SD (MD −2.02; 95% CI −3.45 to −0.59; *n* = 90), and night diastolic SD (MD −1.54; 95% CI −2.62 to −0.46; *n* = 90). 

*(2) CV after Treatment*. No noteworthy statistical differences of this endpoint in any of the trials were noted. 

*(3) BP after Treatment*. There was no significant difference between the experimental and the control groups regarding this outcome which contained 24 h systolic BP, 24 h diastolic BP, day systolic BP, day diastolic BP, night systolic BP, and night diastolic BP. 

*(4) Antihypertensive Effect*. There was no significant difference between the experimental and the control groups regarding this outcome. 

*(5) QOL*. There was also no significant difference between the experimental and the control groups regarding this outcome which was measured by SF-36 health related QOL scale. 

*(6) Nighttime BP Decreased Rate*. It was not reported in this trial. 

*(7) Frequency of Reversed Abnormal Rhythm of BP*. It was not reported in this trial.

### 3.4. Safety

There were 9 trials reporting 50 cases of adverse events. The experimental groups had 18 patients with side reactions, and the control groups had 29 patients with side reactions. Three patients had adverse events but with no specific group information. No significant difference about adverse events was found between two groups. The most commonly reported adverse events in the 9 trials were intestinal disturbance (abdominal distension, nausea, and constipation) [17, 22, 25, 28], ankle edema [17, 27], dizziness [22, 27, 29], palpitation [22], facial flushing [26], and dry cough [19, 24].

## 4. Discussion

There are thirteen RCTs including 1103 participants that were included in this systematic review. From this review, we may be able to speculate that both the TCM and combination therapy have a significant effect in lowering BP, lowering BPV, and adjusting the circadian rhythm. As we known, efficacy of amlodipine besylate tablets in lowering BP variation is better than other western medicines. Most of trials in this paper selected amlodipine besylate tablets as conventional therapy in the compare group of Chinese medicine combined with conventional therapy versus the same conventional therapy. One trial took placebo combined with conventional therapy as control group. According to TCM theories, the treatment is based on syndrome differentiation, and even the same disease could have a variety of syndromes; therefore, the treatments could be modified with different Chinese herbal medicines. Although the differences in TCM prescription, forms, course of treatment, and control drugs prevent us from performing meta-analysis which could provide precise effect estimate of intervention, from Table 4 we gladly found that there were two kinds of TCM (Ramulus Uncariae Rhynchophyllae cum Uncis and Radix Achyranthis Bidentatae) that appeared most. The most TCM syndromes were yin deficiency and yang excess. This is also consistent with the theory of TCM. It was confirmed that the integrative medicine based on syndrome differentiation, a person-centered and balanced medicine [30], was safer and more effective than western medicine alone in the treatment of hypertension [31]. Adverse reaction related to Chinese medicine to reduce BPV is relatively rare. Only a few patients had ankle edema, dizziness, and nausea during the treatment.

It is too early to recommend this conclusion to clinical practitioners considering the limitations of this review. Firstly, the methodological quality of included trials in the review generally needs to be improved. Studies have shown that the degree of rigorous design and quality of methodology of the study have a direct impact on the effectiveness of intervention [32, 33]. The randomization was unclear in most of trials. Only five RCTs described random allocation in detail, and two RCTs mentioned random hidden method. Blinding is an effective way to control measurement bias; however, only one trial mentioned double-blind; no trial reported estimation of sample size. Moreover, this review focuses on short-term BPV and all of the included trials did not have follow-up research, so we cannot evaluate long-term effect of Chinese herbal medicine for BPV. In addition, all the trials did not publish their protocols, so we can only judge their reporting bias by a compromise method, which is comparing whether the outcomes mentioned in method and results are consistent. Two trials reported QOL, but only one trial evaluated QOL by certain scale. Fifth, due to the clinical features of hypertension, hypertensive patients commonly also suffered from other diseases, such as coronary heart disease or hyperlipidemia, but only a small number of clinical trials on the baseline data reported these accompanied diseases. Most of the other tests did not report this information, but we cannot rule out the possibility of patients with accompanied disease. Sixth, we only included 13 trials with a relative small sample size in this review, and we failed to perform meta-analysis. Therefore, further rigorously designed RCTs are needed before recommending TCM to patients with hypertension. Moreover, we suggest that the design and reporting of RCTs on TCM strictly comply with both CONSORT statement [34] and that for herbal interventions [35].

## 5. Conclusion

TCM showed potentially short-term beneficial effects on BPV. However, because of small sample size and potential bias of most trials, this result should be interpreted with caution. More high quality trials, safety evidence, and long-term effects are warranted before TCM is recommended as an alternative or complementary medicine for BPV in hypertension patients. Information of QOL and economic effectiveness should also be paid more attention in future clinical trials.

## Figures and Tables

**Table 1:** Search strategy.

(1) Cochrane Central Register of Controlled Trials (CENTRAL) on The Cochrane Library	
#1 MeSH descriptor: [Medicine, Chinese Traditional] explode all trees	
#2 MeSH descriptor: [Drugs, Chinese Herbal] explode all trees	
#3 #1 or #2	
#4 MeSH descriptor: [Hypertension] explode all trees	
#5 “standard deviation” or SD	
#6 “coefficient of variability” or CV	
#7 #5 or #6	
#8 #4 and #7	
#9 “blood pressure variability” or “BPV” or “blood pressure fluctuation”	
#10 “Randomized Controlled Trial” or “RCT”	
#11 #8 or #9	
#12 #3 and #10 and #11	

(2) PubMed	
#1 “Hypertension” [MeSH]	
#2 “medicine, Chinese traditional” [MeSH Terms]	
#3 “drugs, Chinese herbal” [MeSH Terms]	
#4 #2 OR #3	
#5 “randomized controlled trial” [All Fields] or RCT [All Fields]	
#6 BPV [All Fields] OR “blood pressure variability” [Title/Abstract] OR “blood pressure fluctuation” [Title/Abstract]	
#7 “standard deviation” [Title/Abstract] or SD [All Fields]	
#8 “coefficient of variability” [Title/Abstract] or CV [All Fields]	
#9 #7 OR #8	
#10 #1 AND #9	
#11 #6 OR #10	
#12 #4 AND #11	
#13 choose filter “Randomized Controlled Trial”	

(3) CBM	
(((“blood pressure variability” [Common fields: intelligence]) OR (“fluctuation of blood pressure” [Common fields: intelligence]) OR (“BPV” [Common fields: intelligence]) OR (“blood pressure variability” [Common fields: intelligence])) AND (“randomized” [All: intelligence]) AND ((“Chinese medicine” [All: intelligence]) OR (“Chinese patent medicine” [All: intelligence]) OR (“integrated Chinese and western medicine” [All: intelligence]) OR (“granule” [All: intelligence]) OR (“Chinese herbal medicine” [All: intelligence]) OR (“traditional Chinese medicine” [All: intelligence])))	

(4) CNKI	
(SU = “blood pressure variability” OR SU = “fluctuation of blood pressure” OR SU = “BPV”) AND (FT = “traditional Chinese medicine” OR FT = “Chinese patent medicine” OR FT = “integrated Chinese and western medicine” OR FT = “granule” OR FT = “Chinese herbal medicine” OR FT = “Chinese medicine”) AND (FT = “randomized”)	

(5) VIP	
(Title or key word = blood pressure variability + fluctuation of blood pressure + BPV) and (Any Field = traditional Chinese medicine + integrated Chinese and western medicine + granule + Chinese patent medicine + Chinese herbal medicine + Chinese medicine) and (Any Field = randomized) and (Professional = pharmaceutical and health care) and (Range = all journals)	

(6) WanFang database	
(title or key word: (blood pressure variability) + title or key word: (fluctuation of blood pressure) + title or key word: (BPV)) ∗ (Chinese medicine + Chinese patent medicine + integrated Chinese and western medicine + granule + Chinese herbal medicine + traditional Chinese medicine) ∗ randomized	

**Table 2:** Basic characteristics of included studies.

Study ID	Source of participants	Sample size (I/C)	Age (years, I/C)	Sex (M/F)	Intervention	Control	Treatment duration (week or month)	Outcomes
Pan 2012 [17]	Outpatients and inpatients	I: 30 C: 30	I: 66.5 ± 4.5 (skewed distribution) C: 67 ± 6.25 (Skewed distribution)	NR	Tian ma shu xin granule, 150 mL bid	Amlodipine besylate tablet, 5 mg qd	4 w	24 hCV, dCV, nCV 24 hSD, dSD, nSD 24 hBP, dBP, nBP

Ma 2013 [18]	Outpatients and inpatients	I: 20 C: 20	I: 49.00 ± 11.82 C: 55.10 ± 10.44	21/19	Qian yang yu yin granule, 10 g tid	Combined felodipine with enalapril to control blood pressure below 140/90 mmHg. felodipine, 5 mg/d, enalapril 10 mg/d, dose adjustment if blood pressure is above 140/90 mmHg	8 w	24 hSD, dSD, nSD 24 hBP, dBP, nBP

Liang 2007 [19]	Outpatients and inpatients	I: 31 C: 30	I: 50.39 ± 8.00 C: 51.60 ± 7.07	36/25	Qin dan capsule, 1.75 g tid	Enalapril maleate tablet, 5 mg bid group	8 w	dSD, nSD 24 hBP, The nighttime blood pressure decrease rate

Shi 2011 [20]	Outpatients and inpatients	I: 20 C: 20	I: 74.25 ± 8.42 C: 73.05 ± 9.32	33/7	Sang ji wen dan decoction, one packet, bid	Amlodipine besylate tablet, 5 mg qd, group	4 w	24 hSD, dSD, nSD 24 hBP, dBP, nBP

Wang 2011 [21]	Outpatients	I: 70 C: 68	I: 52.1 ± 9.3 C: 50.3 ± 8.9	73/65	Songling Xuemaikang capsule, 1.5 g, tid	Amlodipine besylate tablet, 5 mg qd	4 w	dSD, nSD 24 hBP

Kang and Fan 2013 [22]	Outpatients and inpatients	I: 60 C: 58	I: 67 ± 10 C: 66 ± 9	72/46	Xian dan tong mai decoction, one packet, bid	Amlodipine besylate tablet, 5 mg qd	4 w	24 hSD, dSD, nSD 24 hBP, dBP, nBP

Ma et al. 2013 [23]	Outpatients	I: 41 C: 37	I: 50.42 ± 4.34 C: 51.45 ± 4.70	NR	Xiang tian ma decoction, one packet, bid	Benazepril, 10 mg qd	8 w	24 hSD, dSD, nSD 24 hBP, dBP, nBP

Li 2012 [24]	NR	I: 54 C: 56	I: NR C: NR	NR	Xuezhikang capsule 600 mg, bid	A combination of ACEI and CCB as basic treatment, drug adjustment according to the circumstances	6 m	24 hSD 24 hBP

Ji and Han 2011 [25]	Outpatients and patients in hospital	I: 80 C: 80	I: 53.3 ± 11.08 C: 56.7 ± 11.61	90/70	Yang xue qing nao granule, one packet, tid	Conventional antihypertensive drugs	12 w	dSD, nSD 24 hBP, dBP, nBP, Circadian rhythm of blood pressure

Zhang et al. 2012 [26]	Outpatients and patients in hospital	I: 40 C: 40	I: NR C: NR	NR	Yin gan jing decoction, one packet, bid	Amlodipine besylate tablet, 5 mg qd, group	1 m	24 hSD, 24 hBP, dBP, nBP, trough-to-peak ratios, 24 h smoothness index (SI) The nighttime blood pressure decreased rate

Yan 2008 [27]	Outpatients and patients in hospital	I: 30 C: 28	I: 61.54 ± 6.53 C: 60.94 ± 8.01	30/28	Tiao ping kang tablet 4 tablets, tid	Nifedipine controlled released Tablets, 30 mg, qd	4 w	24 hSD, dSD, nSD 24 hBP, dBP, nBP, trough-to-peak ratios, Circadian rhythm of blood pressure, lowering blood pressure load value, morning blood pressure, stationarity index pulse pressure, pulse pressure drop rate

Liang 2011 [28]	NR	I: 35 C: 35	I: 61.3 ± 5.38 C: 61.7 ± 4.76	32/38	Gou teng si wu decoction, one packet, bid	Levamlodipine besylate tablets, 2.5 mg, qd	4 w	24 hSD, 24 hBP, 24 h blood pressure load, 24 h trough-to-peak ratios the nighttime blood pressure decrease rate

Chen 2012 [29]	Outpatients	I: 45 C: 45	I: NR C: NR	NR	Qing xuan granule, one packet, bid	Amlodipine besylate tablet, 5 mg qd, group	8 w	24 hCV, dCV, nCV 24 hSD, dSD, nSD 24 hBP, dBP, nBP

Notes: I: intervention group; C: control group; NR: not reported, that is, no related information is reported in the articles.

RCT: randomized controlled trial; qd: once a day; bid: twice a day; tid: three times a day; w: week; m: month; CV: coefficient of variability; SD: standard deviation; BP: blood pressure; h: hour; d: day; n: night.

**(a):** 

Intervention versus control	Effect estimate of outcomes (RR or MD (95% CI))											
	24 hsSD	24 hdSD	dsSD	ddSD	nsSD	ndSD	2 hsCV	24 hdCV	dsCV	ddCV	nsCV	ndCV
(1) Chinese medicine combined with conventional therapy versus the same conventional therapy												
Xiandan tongmai decoction [22]	−2.40 [−3.50, −1.30]	−1.60 [−3.12, −0.08]	−1.40 [−2.63, −0.17]	−1.90 [−3.28, −0.52]	−1.40 [−2.63, −0.17]	0.50 [−1.10, 2.10]	NR	NR	NR	NR	NR	NR
Xuezhikang capsule [24]	−1.79 [−2.79, −0.79]	−0.72 [−1.97, 0.53]	NR	NR	NR	NR	NR	NR	NR	NR	NR	NR
Gouteng siwu decoction [28]	0.28 [−1.31, 1.88]	−0.21 [−1.31, 0.89]	NR	NR	NR	NR	NR	NR	NR	NR	NR	NR
Xiang tian ma decoction [23]	−5.20 [−6.37, −4.03]	−0.67 [−1.59, 0.25]	−4.56 [−5.85, −3.27]	−1.14 [−2.09, −0.19]	−6.18 [−7.74, −4.62]	−1.19 [−2.14, −0.24]	NR	NR	NR	NR	NR	NR
Qianyang yuyin granule [18]	−1.04 [−2.74, 0.66]	−0.40 [−1.64, 0.84]	−1.47 [−3.39, 0.45]	−0.54 [−1.96, 0.88]	−0.89 [−3.85, 2.07]	−0.84 [−2.69, 1.01]	NR	NR	NR	NR	NR	NR
Tianma shuxin granule [17]	−1.24 [−2.65, 0.17]	−0.04 [−1.11, 1.03]	−1.06 [−2.30, 0.18]	−0.43 [−1.64, 0.78]	−0.37 [−2.27, 1.52]	−1.29 [−3.18, 0.60]	−0.00 [−0.01, 0.01]	0.03 [0.02, 0.04]	−0.01 [−0.02, −0.00]	−0.01 [−0.02, 0.01]	−0.01 [−0.02, 0.01]	−0.01 [−0.03, 0.01]
Sangji wendan decoction [20]	−2.44 [−4.38, −0.50]	−2.13 [−4.34, 0.08]	−0.02 [−2.21, 2.17]	−1.74 [−4.14, 0.66]	−1.00 [−3.33, 1.33]	−1.78 [−4.34, 0.78]	NR	NR	NR	NR	NR	NR
Tiao ping kang tablet [27]	−0.40 [−3.30, 2.50]	−0.62 [−3.72, 2.48]	−3.87 [−6.16, −1.58]	−4.95 [−7.10, −2.80]	−5.29 [−7.40, −3.18]	−1.82 [−4.09, 0.45]	NR	NR	NR	NR	NR	NR
Yin gan jing decoction [26]	−0.90 [−1.45, −0.35]	−0.24 [−0.46, −0.02]	NR	NR	NR	NR	NR	NR	NR	NR	NR	NR
Yangxue qingnao granule [25]	NR	NR	−0.50 [−0.81, −0.19]	−0.15 [−0.50, 0.20]	−0.39 [−0.60, −0.18]	−0.21 [−0.36, −0.06]	NR	NR	NR	NR	NR	NR
Qin dan capsule [19]	NR	NR	−2.15 [−3.39, −0.91]	−2.20 [−3.26, −1.14]	−1.15 [−2.21, −0.09]	−1.40 [−2.51, −0.29]	NR	NR	NR	NR	NR	NR
Songling Xuemaikang capsule [21]	NR	NR	−0.70 [−1.48, 0.08]	−0.60 [−1.08, −0.12]	−1.60 [−2.19, −1.01]	−0.10 [−0.59, 0.39]	NR	NR	NR	NR	NR	NR

(2) Chinese medicine combined with conventional therapy versus placebo combined with the same conventional therapy												
Qing xuan granule [29]	−1.47 [−2.52, −0.42]	−1.26 [−1.93, −0.59]	−1.60 [−2.65, −0.55]	−1.02 [−1.70, −0.34]	−2.02 [−3.45, −0.59]	−1.54 [−2.62, −0.46]	0.00 [−0.01, 0.01]	−0.01 [−0.07, 0.05]	0.00 [−0.01, 0.02]	0.00 [−0.01, 0.01]	−0.01 [−0.02, 0.01]	−0.01 [−0.03, 0.01]

**(b):** 

Intervention versus control	Effect estimate of outcomes (RR or MD (95% CI))											
	24 hsBP	24 hdBP	dsBP	ddBP	nsBP	ndBP	AE^∗^	Adverse effect^∗^	Quality of life	NSBPDR	NDBPDR	RARBPR^∗^
(1) Chinese medicine combined with conventional therapy versus the same conventional therapy												
Xiandan tongmai decoction [22]	−0.20 [−3.23, 2.83]	0.10 [−2.66, 2.86]	−1.70 [−4.93, 1.53]	−0.40 [−3.40, 2.60]	1.10 [−1.99, 4.19]	0.10 [−2.66, 2.86]	0.59 [0.22, 1.57]	NR	NR	NR	NR	NR
Xuezhikang capsule [24]	−5.00 [−8.80, −1.20]	−1.00 [−3.07, 1.07]	NR	NR	NR	NR	NR	0.80 [0.29, 2.21]	NR	NR	NR	NR
Gouteng siwu decoction [28]	−14.65 [−20.29, −9.01]	−4.05 [−8.09, −0.01]	NR	NR	NR	NR	0.32 [0.10, 1.04]	3.09 [0.12, 78.41]	NR	NR	NR	NR
Xiang tian ma decoction [23]	−2.90 [−6.27, 0.47]	−4.05 [−7.23, −0.87]	3.50 [0.42, 6.58]	−3.72 [−5.23, −2.21]	−6.80 [−12.85, −0.75]	−0.75 [−3.29, 1.79]	NR	0.25 [0.05, 1.26]	NR	NR	NR	NR
Qianyang yuyin granule [18]	−9.60 [−13.49, −5.71]	−6.45 [−10.58, −2.32]	−9.85 [−14.14, −5.56]	−0.84 [−2.69, 1.01]	−7.80 [−13.04, −2.56]	−6.40 [−10.72, −2.08]	NR	NR	NR	NR	NR	NR
Tianma shuxin granule [17]	−0.20 [−3.67, 3.27]	1.73 [−2.83, 6.29]	2.00 [−1.73, 5.73]	0.73 [−3.08, 4.54]	−5.84 [−15.76, 4.08]	1.46 [−2.37, 5.29]	NR	1.00 [0.19, 5.40]	NR	−0.01 [−0.02, 0.01]	−0.01 [−0.03, 0.01]	NR
Sangji wendan decoction [20]	0.52 [−6.65, 7.69]	0.16 [−4.47, 4.79]	−0.35 [−8.01, 7.31]	−0.42 [−5.50, 4.66]	1.46 [−7.06, 9.98]	0.63 [−4.75, 6.01]	0.32 [0.01, 8.26]	NR	NR	NR	NR	NR
Tiao ping kang tablet [27]	−0.10 [−4.47, 4.27]	−1.00 [−4.95, 2.95]	−3.40 [−7.38, 0.58]	−2.90 [−5.73, −0.07]	−8.70 [−13.18, −4.22]	−4.00 [−6.81, −1.19]	0.93 [0.17, 5.02]	0.09 [0.00, 1.74]	NR	NR	NR	0.17 [0.04, 0.66]
Yin gan jing decoction [26]	−2.92 [−5.51, −0.33]	−2.57 [−4.68, −0.46]	−3.15 [−5.66, −0.64]	−2.40 [−4.39, −0.41]	−3.77 [−6.39, −1.15]	−3.52 [−5.85, −1.19]	0.30 [0.09, 0.93]	0.19 [0.01, 4.09]	NR	NR	NR	NR
Yangxue qingnao granule [25]	−4.90 [−7.10, −2.70]	−2.11 [−3.60, −0.62]	−3.55 [−5.65, −1.45]	−1.20 [−2.14, −0.26]	−4.76 [−7.77, −1.75]	−2.68 [−4.82, −0.54]	NR	5.13 [0.24, 108.51]	NR	NR	NR	0.37 [0.15, 0.94]
Qin dan capsule [19]	−4.61 [−8.88, −0.34]	−0.34 [−3.05, 2.37]	NR	NR	NR	NR	0.45 [0.08, 2.65]	0.62 [0.10, 4.00]	NR	NR	NR	NR
Songling Xuemaikang capsule [21]	−6.10 [−9.73, −2.47]	−9.80 [−12.62, −6.98]	NR	NR	NR	NR	NR	NR	NR	NR	NR	NR

(2) Chinese medicine combined with conventional therapy versus placebo combined with the same conventional therapy												
Qing xuan granule [29]	−1.15 [−6.17, 3.87]	−2.24 [−6.66, 2.18]	−0.91 [−6.33, 4.51]	−2.11 [−6.76, 2.54]	0.53 [−4.83, 5.89]	−1.69 [−5.92, 2.54]	0.51 [0.18, 1.44]	NR	−34.07 [−55.89, −12.25]	NR	NR	NR

AE: antihypertensive effect.

NSBPDR: nighttime systolic blood pressure decreased rate.

NDBPDR: nighttime diastolic blood pressure decreased rate.

RARBPR: reversed abnormal rhythm of blood pressure rate.

NR: not reported, the article did not report any information about this item.

NO: not observed, the article reported that no adverse effects were observed in the studies.

Outcomes marked ∗ were MD; others were RR.

**Table 4:** Herbal medicines and adverse effects in the included trials.

Name of herbal medicines	Formulation	Compositions	Adverse events	Study ID
Xiandan tongmai	Decoction	Herba Epimedii Brevicornus 15 g, Rumulus Ginnamomi 10 g, Radix Salviae Miltiorrhizae 15 g, Fructus Macrocarpii 10 g, Fructus Lycii 10 g, Herba Taxilli Chinensis 10 g, Cortex Eucommiae 10 g, Radix Notoginseng 3 g, Radix Angelicae Sinensis 10 g, Rhizoma Chanxiong 10 g, Rhizoma Anemarrhenae 6 g, Cortex Phellodendri Amurensis 6 g, Radix Achyranthis Bidentatae 10 g	Two patients felt gastrointestinal discomfort and sick in treatment group, but symptoms disappeared with medication after meal instead	Kang and Fan 2013 [22]

Xuezhikang	Capsule	Ultivarietas Oryzae Sativae et Monasci (dosage not available)	Not reported	Li 2012 [24]

Gouteng siwu	Decoction	Herba Taxilli Chinensis 12 g, Radix Achyranthis Bidentatae 12 g, Ramulus Uncariae Rhynchophyllae cum Uncis 30 g, Concha Haliotidis 20 g, Rhizoma Gastrodiae 15 g, Rhizoma Chanxiong 15 g, Rhizoma et Radix Notopterygii 6 g, Radix Rehmanniae 15 g, Radix Paeoniae Alba 15 g	One patient had mild nausea and felt epigastric discomfort for two days, but symptoms were relieved by themselves without treatment or stopping medication	Liang 2011 [28]

Xiang tian ma	Decoction	Ziziphora Clinopodioides Lam 12 g, Radix et Rhizoma Rhodiolae Kirilowii 12 g, Radix Rehmanniae 12 g, Flos Rosae Rugosae 12 g, Apocynum venetum L 20 g, and so on	Not observed	Ma et al. 2013 [23]

Qianyang yuyin	Decoction	Herba Bidentis Bipinnatae, Radix Scrophulariae, Fructus Macrocarpii, Radix Polygoni Multiflori, Rhizoma Alismatis, Radix Achyranthis Bidentatae (dosage not available)	Not reported	Ma 2013 [18]

Tianma shuxin	Granule	Rhizoma Gastrodiae, Radix Achyranthis Bidentatae, Concha Ostreae, Radix Paeoniae Rubra, Cocculus orbiculatus (L.) DC, Rhizoma Alismatis, Radix Scrophulariae, Hirudo (dosage not available)	In treatment group, one had mild nausea, one had epigastric fullness, and one had increased stool frequency, but symptoms disappeared after 3-4 days without drug withdrawal	Pan 2012 [17]

Sangji wendan	Decoction	Folium Mori 10 g, Fructus Tribuli 10 g, Rhizoma Gastrodiae 10 g, Dendranthema lavandulifolium (Fisch. ex Trautv.) Ling et Shih 10 g, Ramulus Uncariae Rhynchophyllae cum Uncis 12 g, Concha Haliotidis 30 g, Pericarpium Citri Reticulatae 6 g, Rhizoma Pinelliae (processed with ginger) 10 g, Fructus Aurantii Immaturus 10 g, poria 12 g, Fructus et Semen Trichosanthis Kirilowii 12 g, Caulis Bambusae in Taeniam 6 g	Not observed	Shi 2011 [20]

Tiao ping kang	Tablet	Fructus Ligustri Lucidui, Herba Epimedii Brevicornus, Caulis et Folium Polygoni Multiflori, Herba Leonuri Japonici, ilex pubescens hook. et arn. and so on (dosage not available)	Not reported	Yan 2008 [27]

Yin gan jing	Decoction	Ramulus Uncariae Rhynchophyllae cum Uncis 20 g, Fructus Evodiae Rutaecarpae 15 g	Not reported	Zhang et al. 2012 [26]

Yangxue qingnao	Granule	Radix Angelicae Sinensis, Rhizoma Chanxiong, Ramulus Uncariae Rhynchophyllae cum Uncis, Radix Paeoniae Alba, Concha Margaritifera Usta, Spica Prunellae Vulgaris, Radix Rehmanniae preparata, Semen Cassiae Obtusifoliae, and so on (dosage not available)	Two patients felt stomach discomfort: symptoms disappeared after medication after meal	Ji and Han 2011 [25]

Qin dan	Capsule	Radix Scutellariae Baicalensis, Rhizoma Coptidis, Ramulus Uncariae Rhynchophyllae cum Uncis, Radix Salviae Miltiorrhizae, Rhizoma Chanxiong, Pheretima Aspergillum, Herba Leonuri Japonici, Herba Taxilli Chinensis (dosage not available)	Not reported	Liang 2007 [19]

Songling Xuemaikang	Capsule	Radix Puerariae Lobatae, Margarita, Folium Pini Massonianae (dosage not available)	Not reported	Wang 2011 [21]

Qing xuan	Granule	Rhizoma Gastrodiae 30 g, Folium Ilicis Cornutae Immaturum 30 g, Cortex Eucommiae 30 g, Radix Scutellariae Baicalensis 15 g, Folium Ilicis Cornutae Immaturum 15 g	Two patients complained of dizziness and then stopped taking this drug. One had lobar pneumonia and then took other medicines. Four cases were lost to follow-up	Chen 2012 [29]

Not reported: the article did not report any information about adverse effects. Not observed: the article reported that no adverse effects were observed in the studies.
